# A familial case of interstitial deletion of the short arm of chromosome 6p22.3-p24.3 in twins with severe delay in psychomotor and speech development

**DOI:** 10.18699/vjgb-25-70

**Published:** 2025-09

**Authors:** G.D. Moskvitin, D.B. Kochkina, M.K. Gurinova, D.A. Fedotov, L.V. Bekenieva, A.A. Kashevarova, A.L. Sukhomyasova, I.N. Lebedev, N.R. Maximova

**Affiliations:** M.K. Ammosov North-Eastern Federal University, Yakutsk, Russia M.E. Nikolaev Republic Hospital No. 1 – National Center of Medicine, Yakutsk, Russia; M.K. Ammosov North-Eastern Federal University, Yakutsk, Russia M.E. Nikolaev Republic Hospital No. 1 – National Center of Medicine, Yakutsk, Russia; Ammosov North-Eastern Federal University, Yakutsk, Russia; Research Institute of Medical Genetics, Tomsk National Research Medical Center of the Russian Academy of Sciences, Tomsk, Russia; M.K. Ammosov North-Eastern Federal University, Yakutsk, Russia M.E. Nikolaev Republic Hospital No. 1 – National Center of Medicine, Yakutsk, Russia; Research Institute of Medical Genetics, Tomsk National Research Medical Center of the Russian Academy of Sciences, Tomsk, Russia; M.K. Ammosov North-Eastern Federal University, Yakutsk, Russia; Research Institute of Medical Genetics, Tomsk National Research Medical Center of the Russian Academy of Sciences, Tomsk, Russia; M.K. Ammosov North-Eastern Federal University, Yakutsk, Russia

**Keywords:** interstitial deletion 6p22.3-p24.3, intellectual disorders, psychomotor and speech delay, autism spectrum disorder, microarray comparative genomic hybridization, интерстициальная делеция 6p22.3-p24.3, интеллектуальные расстройства, задержка психо-речевого развития, расстройство аутистического спектра, микроматричная сравнительная геномная гибридизация

## Abstract

Interstitial deletions of the short arm of chromosome 6 are even rarer than distal deletions of 6p24-pter, with an incidence rate of 1:1,000,000 (according to MalaCards, https://www.malacards.org/). These deletions are associated with developmental delays, autism spectrum disorders, congenital anomalies, and dysmorphic features. The objective of our study was to identify chromosomal abnormalities in twins from a Yakut family exhibiting severe psycho-speech developmental delays, intellectual disability combined with dysmorphisms, and congenital anomalies. In this paper, two new cases involving monozygotic twins from a Yakut family, who underwent array comparative genomic hybridization (aCGH), were reported. The diagnostic results revealed a rare interstitial deletion in the region 6p22.3-p24.3, measuring 7.5 Mb, which was subsequently confirmed using a conventional cytogenetics (GTG-banding) method. According to the cytogenetic analysis, the karyotypes of the parents were normal, indicating a de novo structural chromosomal rearrangement in the patients. Additionally, a comparative phenotypic analysis of these twins with each other and with other previously reported patients was performed; they were found to have overlapping deletions in the 6p22-p24 region. Furthermore, a literature review and an analysis of the gene content of the deleted region 6p22.3-p24.3 were conducted, and so was a discussion of the genotype-phenotype correlation. The results of the phenotypic analysis revealed both common and distinct dysmorphogenic features, including craniofacial dysmorphisms, deformities of the auricles, and abnormalities in the development of the upper and lower limbs, which are often mentioned in the literature. However, the analyzed data, both from the literature and our observations, showed that all patients lacked a common deleted region in the 6p22-p24 area, creating challenges in establishing an accurate diagnosis. The findings indicate the complexity of defining the minimally overlapping region responsible for the observed phenotypic and behavioral traits and highlight the importance of a systematic and multi-level approach to diagnosing severe psycho-speech developmental delays.

## Abstract (ru)

Интерстициальные делеции короткого плеча хромосомы 6 встречаются еще реже, чем дистальные делеции 6p24-pter с частотой 1:1 000 000 (по данным MalaCards, https://www.malacards.org/), и ассоциируются с задержками развития, расстройствами аутистического спектра, врожденными аномалиями, а также дисморфическими особенностями. Цель нашего исследования заключалась в поиске хромосомной патологии у близнецов из якутской семьи с грубой задержкой психо-речевого развития, умственной отсталостью в сочетании с дисморфиями и врожденными аномалиями. В этой работе мы сообщаем о двух новых пациентах – монозиготных близнецах из одной якутской семьи, которым была проведена микроматричная сравнительная геномная гибридизация (aCGH). В результате диагностики обнаружена редкая интерстициальная делеция в регионе 6p22.3-p24.3 размером 7.5 Мб, которая ретроспективно была подтверждена анализом GTG – дифференциального окрашивания хромосом. По данным цитогенетического исследования, кариотипы родителей были нормальными, что говорит о de novo структурной хромосомной перестройке у пациентов. Также мы выполнили сравнительный фенотипический анализ этих близнецов между собой и с другими ранее описанными в литературе пациентами, у которых были найдены перекрывающиеся делеции в регионе 6р22-р24. Кроме того, проведены обзор литературы и анализ генного состава делетированного региона 6p22.3-p24.3 с обсуждением корреляции генотип-фенотип. По результатам фенотипического анализа выявлены как общие, так и различные стигмы дизморфогенеза, такие как краниофациальные дисморфии, деформации ушных раковин и отклонения в развитии верхних и нижних конеч- ностей, часто упоминаемые в литературе. Однако в проанализированных данных как в литературе, так и в наших наблюдениях у всех пациентов отсутствовал общий делетированный регион в области 6p22-p24, что создает трудности в установлении точного диагноза. Полученные результаты указывают на сложность однозначного определения минимально перекрывающегося региона, ответственного за наблюдаемые фенотипические и поведенческие особенности, и на важность последовательного и многоуровневого подхода к диагностике грубой задержки психо-речевого развития.

## Introduction

The frequency of intellectual disorders (ID) in the world is 2–3 % (McKenzie et al., 2016); 1–3 % of children suffer from delayed psychomotor development combined with dysmorphia and congenital anomalies (Shaffer, 2005). It is known that the proportion of children with disabilities due to mental and behavioral disorders in Russia reaches 31 % (Freize et al., 2025). Genetic factors account for 17–47 % of the causes of intellectual disabilities (Moeschler, Shevell, 2006). Aneuploidies, large deletions and duplications, and unbalanced chromosomal translocations occur in 30–35 % of patients with intellectual disabilities and, as a rule, underlie syndromic forms of intellectual disability (Willemsen, Kleefstra, 2014).

Deletions affecting the distal part of the short arm of chromosome 6 are relatively rare. According to the MalaCards website (https://www.malacards.org/), the frequency of 6p24- pter chromosome deletion syndrome in the population is less than 1 per 1,000,000 people. Distal deletions of 6p24-pter are associated with developmental delay, brain malformations (including Dandy–Walker malformation, MIM 220200), anterior chamber abnormalities, hearing loss, ear abnormalities, micrognathia, and heart defects (Mirza et al., 2004). Patients with larger 6p23-pter deletions also have microcephaly, genital abnormalities, speech disorders, and delayed motor development (Plaja et al., 1994; Celestino-Soper et al., 2012). Interstitial deletions on 6p22-p24 are registered even less frequently and are usually associated with delayed psychomotor development and growth, hypotension, as well as a number of congenital anomalies, including hydrocephalus, microcephaly, structural eye abnormalities, hypertelorism, low-set and deformed ears, nasal anomalies, micrognathia, palate anomalies, short neck with folds on the skin, heart defects, kidneys and feet, abnormal genitals and abnormal fingers with nail hypoplasia (Plaja et al., 1994; Mirza et al., 2004; Celestino-Soper et al., 2012).

There are two reports in the scientific literature about interstitial deletion on chromosome 6p22.3-p24.3. In one of them, the authors used microarray comparative genomic hybridization (aCGH) to identify a ~5.4 Mb deletion on chromosome 6p22.3-p23 in a 15-year-old patient with intellectual disability and autism spectrum disorder (ASD) (Celestino-Soper et al., 2012). They suggest that the cause of developmental delay and ASD is related to the deletion of the ATXN1, DTNBP1, JARID2, and NHLRC1 genes. The same article describes 17 more patients who had overlapping interstitial deletions on chromosome 6p22-p24. Most patients had neurological or behavioral abnormalities, including developmental and speech delays, ASD, attention deficit hyperactivity disorder (ADHD), repetitive movements, and various dysmorphic facial features.

Another article describes a rare case of interstitial deletion on the short arm of chromosome 6 in a fetus with multiple malformations, detected prenatally by the standard cytogenetic method of amniotic fluid at the 26th week of pregnancy. After termination of pregnancy, the authors eliminated the possibility of insertion of chromosome 6 material into any other chromosome using fluorescent in situ hybridization (FISH) with a full-chromosome probe for chromosome 6 and subtelomeric 6p and 6q probes. Next, molecular karyotyping was performed using the aCGH method, which revealed a rare de novo interstitial deletion 6p22.3-p24.3 (Colmant et al., 2009).

In this study, two new twin patients from the same Yakut family who were diagnosed with a rare de novo interstitial deletion in the 7.5 Mb region 6p22.3-p24.3 are described. Based on the analysis of the previous data, as well as published materials, a comparative phenotypic analysis of these twins between themselves and with other patients with overlapping deletions in the 6p22-p24 region was conducted. A review of the literature and an analysis of the gene composition with a discussion of genotype and phenotype correlations were carried out.

The purpose of the research was to find a chromosomal pathology in twins from a Yakut family who have a severe delay in psycho-speech development and mental retardation.

## Materials and methods

The research was approved by the Committee on Biomedical Ethics of the Scientific Research Institute of Medical Genetics of Tomsk National Research Medical Center (Protocol No. 15 dated 28.02.2023). Informed voluntary consent to participate in the research was received, signed by the parents of the study participants

Clinical, genealogical and cytogenetic studies of the studied family were conducted on the basis of the Medical and Genetic Center of the State Autonomous Institution “RH No. 1 – NCoM named after M.E. Nikolaev” using the resources of the biocollection “DNA Bank of Congenital and Hereditary Pathology and Populations of the Republic of Sakha (Yakutia)”.

Cytogenetic examination (karyotyping) was performed on peripheral blood lymphocytes of the patients with GTGdifferential staining of chromosomes at the level of 550 bands according to generally accepted protocols under a light microscope.

Microarray comparative genomic hybridization (aCGH) was performed using SurePrint G3 Human CGH 8 × 60K microarray (Agilent Technologies, Santa Clara, California, USA) in accordance with the manufacturer’s recommendations based on the Scientific Research Institute of Medical Genetics of the Tomsk National Research Medical Center. Labeling and hybridization of the patient’s DNA and reference DNA (Human Reference DNA, Agilent Technologies) were performed using enzymatic labeling and hybridization protocols (v. 7.5, Agilent Technologies). Array images were obtained using the Agilent SureScan microarray scanner (Agilent Technologies). The data obtained were analyzed using the CytoGenomics (v. 5.3.0.14) software (Agilent Technologies) and publicly available databases of genomic variants: (DGV) (http://projects.tcag.ca/variation), MIM (https://omim.org/), DECIPHER (https://www.deciphergenomics.org/) ClinView Analytics (https://clinical-intelligence.org/services/clinviewanalytics/). The aCGH results were analyzed in accordance with the recommendations of the American Collegium of Medical Genetics and Genomics (ACMG) (Riggs et al., 2020) and the Russian Society of Medical Geneticists (Lebedev et al., 2023).

## Results

The patients, 7-year-old boys from a Yakut family, have been registered at the Medical and Genetic Center of the RH No. 1 – NCoM since 2021 at the age of four with a diagnosis of “Residual organic damage of the central nervous system with severe mental retardation. General speech underdevelopment of level 1. Cerebral palsy, mixed tetraparesis”.

It is known from the medical history that the family had previously applied to the Medical and Genetic Center at the 30th week of pregnancy in connection with the carrying of monochoric diamniotic twins. Ultrasound examination of the fetuses revealed a number of changes: fetus No. 1 had edema of Warton’s jelly, as well as a hydrocele; fetus No. 2 had polyhydramnios and congenital heart disease, including a defect of the interventricular septum and possibly an aortic defect, dilation of the pulmonary artery throughout, a narrow isthmus of the aorta with suspected aortic coarctation. Both fetuses had bradycardia

The obstetric and gynecological medical history of the mother is burdened: the first two pregnancies ended in childbirth on time, the third pregnancy ended in spontaneous miscarriage at the early stages, the fourth was terminated at the request of the mother, the sixth ended in childbirth on time. The patients were born from the fifth pregnancy that was threatened with early termination (see the Figure b). The delivery was performed by caesarean section at 36 weeks of gestation. The Apgar score was 5/7 for both children. The birth weight of patient 1 and patient 2 was 3,030 g (percentile 25.1; SDS 0.67) and 2,845 g (percentile 14.0; SDS 1.08), respectively, the height of both patients was 50 cm (percentile 52.4; SDS 0.06).

**Fig. 1.**
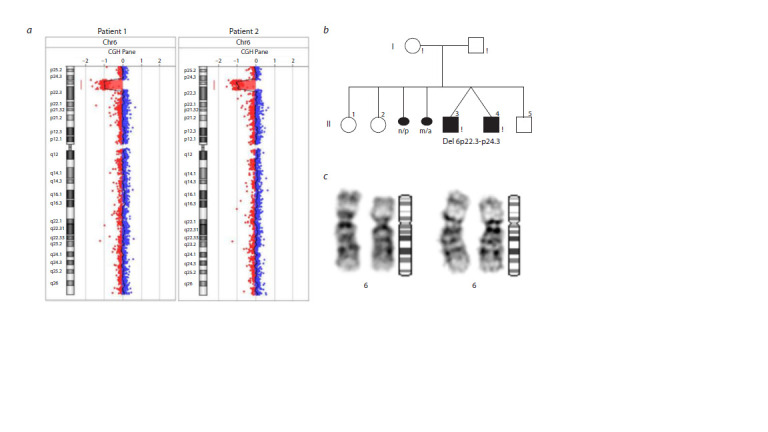
Standard and molecular cytogenetic study, ancestry of the studied family. а – profile of aCGH chromosome 6 in patients 1 and 2; b – family ancestry; n/p – non-developing pregnancy; m/a – medical abortion; c – G-stained chromosomes 6 in patients 1 (left) and 2 (right).

In terms of psychomotor development, both children began to hold their heads at the 2nd month, turn over at the 4th and 5th months, the first child started to sit at the 7th–8th months, the other one first sat at the 9th month. The children started walking with support from the age of one, but at some point both began to lose their acquired skills. The brothers resumed independent walking by the age of two. Among other things, both boys had a delay in speech development, the first words appeared closer to the age of 2 years. However, after the age of three, a regression in psycho-speech development was noted. There is currently no speech. They communicate with pointing gestures and facial expressions, and make inarticulate sounds; if necessary, they lead their relatives by the hand to the object of interest.

Based on the results of the examination and analysis of the phenotypic data of both boys, it is possible to identify both commonalities and differences in their phenotypic characteristics (Table 1).

**Table 1.**
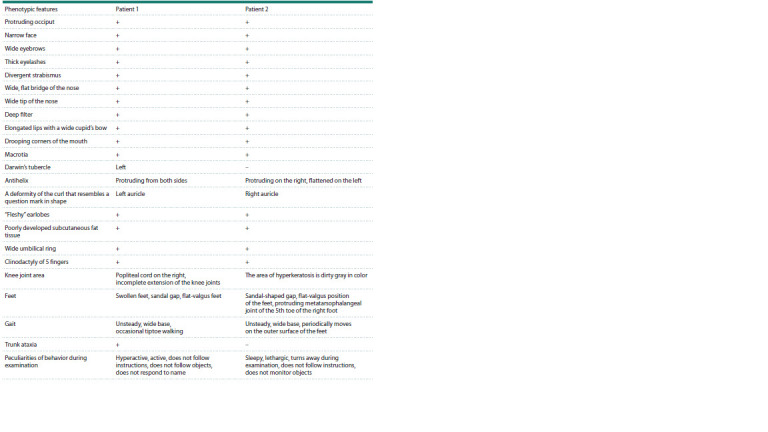
Phenotypic features in twins with deletion 6p22.3-p24.3

In both patients, MRI of the brain with angiography revealed the signs of residual encephalopathy. Based on the examination and assessment of the mental status of the patients, a psychiatrist diagnosed them with “Other organic disorders of behavior and emotions with intellectual and mnestic decline and autistic-like behavior”.

As a result of the aCGH analysis, a pathogenic deletion was detected in the p24.3-p22.3 region of chromosome 6 (arr[GRCh37] 6p24.3p22.3(10514204_17972394)x1; ISCN, 2020) (see the Figure a). This chromosomal rearrangement has a length of 7.5 Mb and was detected in both patients (see the Figure a). 55 genes are localized in the 6p22.3-p24.3 deletion region, among which 11 are pathogenetically significant according to the MIM database (Table 2).

**Table 2.**
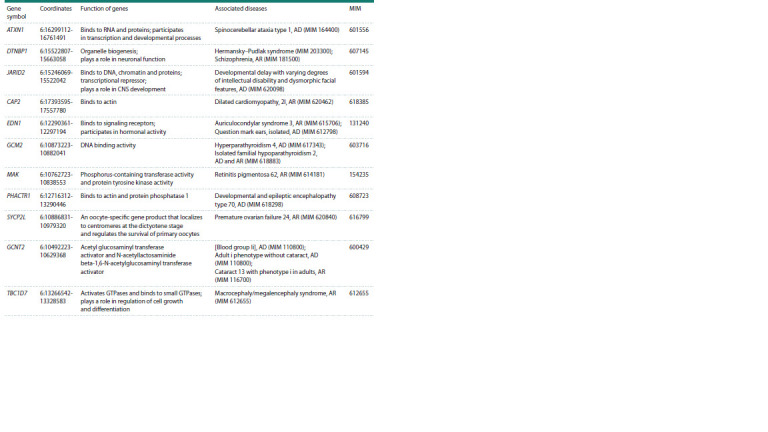
Characteristics of genes located in the region 6p22.3-p24.3 (coordinates: 6:10514204-17972394 at the GRCh37 assembly) Notе. AD – autosomal dominant inheritance; AR – autosomal recessive inheritance. * Gene functions are given based on the Gene Ontology Annotation (http://www.ebi.ac.uk/GOA/).

The standard cytogenetic examination of chromosomes was carried out retrospectively at the Medical and Genetic Center of the State Autonomous Institution “RH No. 1 – NCoM named after M.E. Nikolaev”. As a result, interstitial deletion of 6p22-p24 was confirmed in both patients (see the Figure c). According to the cytogenetic study, the karyotypes of the parents were normal, indicating a de novo structural chromosomal rearrangement in the patients

## Discussion

The interstitial deletion 6p22.3-p24.3 found in Yakut patients in certain regions overlaps with the previously described interstitial deletions in the 6p22-p24 region presented in the scientific literature; however, they have different phenotypic and behavioral features. Search of the databases of diagnostic laboratories Medical Genetics Laboratories (MGL, https:// med-gen.ru/en/) and Signature Genomic Laboratories (SGL, https://www.bionity.com/en/companies/18667/signaturegenomic- laboratories-llc.html) and literary sources revealed 19 more overlapping interstitial deletions, which coincide with the deletion found in our patients with a diagnosis of “Delayed psycho-speech development, mental retardation and autism-like behavior” (Table 3).

**Table 3.**
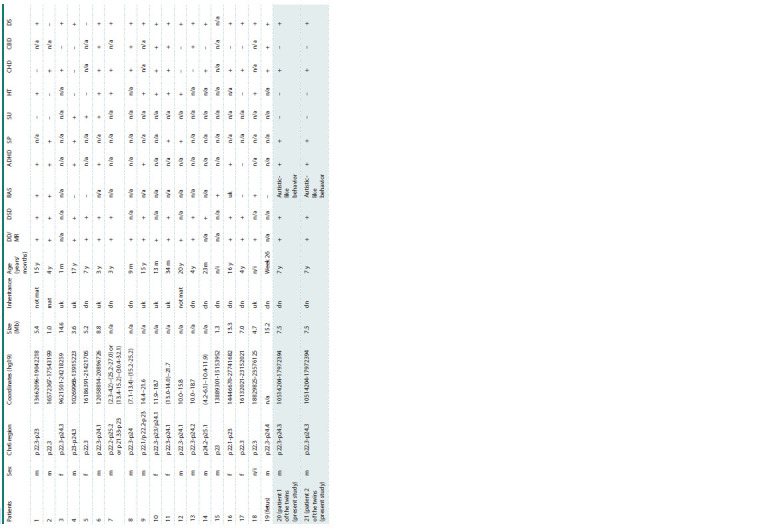
Clinical features of patients with interstitial deletions in the 6p22-p24 region Notе. DD/MR – developmental delay, mental retardation; DSD – delayed speech development; RAS – autism spectrum disorder; ADHD – attention deficit hyperactivity disorder; SP – stereotypical behavior; SU – seizures; HT – hypotension; CHD – congenital heart defects; CBD – congenital brain defect; DS – dysmorphic signs; uk – unknown; n/a (i) – no data; mat – maternal type of inheritance; not mat – non-maternal type of inheritance; dn – de novo. The phenotypic data of patients (1–18) were taken from a scientific article by the author (Celestino-Soper et al., 2005), patient “fetus”, from (Colmant, 2009); the lines highlighted in a darker shade are patients from the present examined family

Out of these 19 patients, 13 (patients 1, 2, 4–7, 9, 11–14, 16, 17) were also diagnosed with ASD and/or manifestations associated with ASD, including delayed speech development, ADHD, and behavioral abnormalities (Table 3) (Tuchman, Rapin, 2022; Goldstein, Schwebach, 2024). Some authors suggest (Celestino-Soper et al., 2012) that some of the following genes may be responsible for ASD traits: ATXN1, JARID2, DTNBP1, and NHLRC1. Some studies have shown that homozygous mice with ATXN1 gene knockout exhibit similar aberrations to transgenic mice of the spinocerebellar ataxia type 1 (SCA1) model with polyglutamine expansions (Matilla et al., 1998; Crespo-Barreto et al., 2010). Despite the absence of ataxic symptoms characteristic of SCA1, as well as progressive cerebellar degeneration in knockout mice, both models showed abnormalities in spatial learning and memory, motor learning and coordination.

In addition, changes in the expression of genes associated with the functional activity of the cerebellum have been reported (Matilla et al., 1998; Crespo-Barreto et al., 2010). It should be noted that in a scientific article (Colmant et al., 2009) described in the literature, cerebellar hypoplasia was recorded in a fetus with deletion 6p22.3-p24.3. In addition, the meta-analysis has shown that single nucleotide polymorphic variants in the ATXN1 gene are associated with IQ in patients with ADHD (Rizzi et al., 2011). A. Bremer and co-authors (2009) hypothesized that ATXN1 haploinsufficiency may contribute to the learning difficulties observed in patients with a 6p22 deletion, which can be noted in our patients who have a deleted 6p22 region (Table 1).

Given the importance of haploinsufficiency for cognitive functions and associations with behavioral abnormalities in mouse models, P.B. Celestino-Soper and co-authors (2012) suggest that heterozygous deletions affecting ATXN1 functionality may be associated with negative consequences of developmental delay and ASD, both in isolation and in combination with other gene deletions.

Deletions in the 6p.24 region are also associated with heart defects. The EDN1 gene (located at 6p24.1) encodes the protein called endothelin-1. A study of the distribution of messenger RNA in various tissues revealed that it is distributed differently in brain and heart tissues. Endothelin has an effect on the central nervous system and on the excitability of neurons. Moreover, the EDN1 gene is involved in both craniofacial and cardiac development (Bogani et al., 2005). Our patients have the same deletion region 6p24.1 as other 10 patients (2, 6–8, 10, 11, 14, 16, 17, 19), who also had congenital heart defects (Table 3), as well as the deleted EDN1 gene. It should be noted that the scientific literature describes a mutation in the endothelin-1 gene that causes auriculocondylar syndrome (MIM 615706), as well as isolated “question mark ears” syndrome (MIM 612798) (Table 2). According to the phenotypic comparison, the patients described by us had similar deformities of the auricles: macrotia, a deformed notch of the outer curl, resembling a “question mark” in shape (Table 1).

The JARID2 gene is expressed in both embryonic and adult human neurons (Berge-Lefranc et al., 1996) and can function as a transcriptional repressor (Toyoda et al., 2003); its mouse homologue, jumonji (Jmj), is necessary for normal neural tube formation and heart development (Takahashi et al., 2004). Patients with a heterozygous deletion in the JARID2 gene, which is assumed to lead to haploinsufficiency of the JARID2 gene, had clinical manifestations of disorders of the nervous system (Barøy et al., 2013; Verberne et al., 2021) (Table 2). The described features in these patients, like in our twins, had characteristic features such as developmental delay, ASD, behavioral disorders, and minor facial phenotype features (Table 1).

In this study, 11 out of 21 patients, including ours, revealed various stigmas of dysmorphogenesis, including craniofacial dysmorphia, ear deformities, and abnormalities in the development of the upper and lower extremities, often mentioned in the literature. In addition, most of the patients had speech disorders and behavioral disorders. However, in the analyzed data, both in the literature and in the present observations, all patients lacked a common deleted region in the 6p22- p24 region, which makes it difficult to establish an accurate diagnosis

## Conclusion

In this research, two new cases of de novo interstitial deletion 6p22.3-p24.3 in monozygotic twins from the same Yakut family were analyzed. After studying the literature, the fact of the rare occurrence of such a deletion in the world was proven. A comparison of the phenotypic and behavioral features between our patients and patients previously described in the literature, who had overlapping deletions in the 6p22-p24 region, was made. In addition to a number of common phenotypic features, differences were found between all patients with deletion in the 6p22-p24 region, including our twins. The phenotypic manifestations caused by variants in certain areas of this region are likely to manifest with incomplete penetrance. This fact may indicate a variation in the severity of traits depending on the presence of modifying factors that may be found in other alleles, regulatory elements, or genes located in different parts of the genome. This makes it difficult to unambiguously identify the minimally overlapping region responsible for the observed phenotypes, and indicates the importance of a consistent and multi-level approach to the diagnosis of severe delayed psycho-speech development.

## Conflict of interest

The authors declare no conflict of interest.
